# Environmental Disturbances Decrease the Variability of Microbial Populations within Periphyton

**DOI:** 10.1128/mSystems.00013-16

**Published:** 2016-05-17

**Authors:** Cristina M. Herren, Kyle C. Webert, Katherine D. McMahon

**Affiliations:** a Freshwater and Marine Sciences Program, University of Wisconsin—Madison, Madison, Wisconsin, USA; b Department of Zoology, University of Wisconsin—Madison, Madison, Wisconsin, USA; c Departments of Bacteriology and Civil and Environmental Engineering, University of Wisconsin—Madison, Madison, Wisconsin, USA; University of Illinois at Urbana-Champaign

**Keywords:** community ecology, disturbance, periphyton, predictability, resilience

## Abstract

A central pursuit of microbial ecology is to accurately model changes in microbial community composition in response to environmental factors. This goal requires a thorough understanding of the drivers of variability in microbial populations. However, most microbial ecology studies focus on the effects of environmental factors on mean population abundances, rather than on population variability. Here, we imposed several experimental disturbances upon periphyton communities and analyzed the variability of populations within disturbed communities compared with those in undisturbed communities. We analyzed both the bacterial and the diatom communities in the periphyton under nine different disturbance regimes, including regimes that contained multiple disturbances. We found several similarities in the responses of the two communities to disturbance; all significant treatment effects showed that populations became less variable as the result of environmental disturbances. Furthermore, multiple disturbances to these communities were often interactive, meaning that the effects of two disturbances could not have been predicted from studying single disturbances in isolation. These results suggest that environmental factors had repeatable effects on populations within microbial communities, thereby creating communities that were more similar as a result of disturbances. These experiments add to the predictive framework of microbial ecology by quantifying variability in microbial populations and by demonstrating that disturbances can place consistent constraints on the abundance of microbial populations. Although models will never be fully predictive due to stochastic forces, these results indicate that environmental stressors may increase the ability of models to capture microbial community dynamics because of their consistent effects on microbial populations.

**IMPORTANCE** There are many reasons why microbial community composition is difficult to model. For example, the high diversity and high rate of change of these communities make it challenging to identify causes of community turnover. Furthermore, the processes that shape community composition can be either deterministic, which cause communities to converge upon similar compositions, or stochastic, which increase variability in community composition. However, modeling microbial community composition is possible only if microbes show repeatable responses to extrinsic forcing. In this study, we hypothesized that environmental stress acts as a deterministic force that shapes microbial community composition. Other studies have investigated if disturbances can alter microbial community composition, but relatively few studies ask about the repeatability of the effects of disturbances. Mechanistic models implicitly assume that communities show consistent responses to stressors; here, we define and quantify microbial variability to test this assumption.

**Author Video**: An author video summary of this article is available.

## Abstract (precis)

There are many reasons why microbial community composition is difficult to model. For example, the high diversity and high rate of change of these communities make it challenging to identify causes of community turnover. Furthermore, the processes that shape community composition can be either deterministic, which cause communities to converge upon similar compositions, or stochastic, which increase variability in community composition. However, modeling microbial community composition is possible only if microbes show repeatable responses to extrinsic forcing. In this study, we hypothesized that environmental stress acts as a deterministic force that shapes microbial community composition. Other studies have investigated if disturbances can alter microbial community composition, but relatively few studies ask about the repeatability of the effects of disturbances. Mechanistic models implicitly assume that communities show consistent responses to stressors; here, we define and quantify microbial variability to test this assumption.

## INTRODUCTION

Many central questions in ecology focus on the sources of variability in populations. Accuracy of predictions is highly valued in ecological studies, and population size is necessarily more predictable when populations are less variable (1, 2). Still, most ecological studies measure changes in the mean number of individuals within populations, rather than the variability of populations across time or space (3, 4). However, the variability of ecological communities is sensitive to environmental drivers and is therefore expected to change in response to disturbances (5, 6). These responses can be observed both temporally, where the variance of a population is calculated over time (7), or spatially, across a landscape or between communities (8). Here, we analyze the variability of populations between replicated microbial communities after a series of experimental disturbances. Specifically, we ask whether this strong environmental forcing creates communities where taxon abundance is less variable than that in undisturbed communities. Thus, we address whether disturbances have repeatable effects on ecological communities.

Some variability naturally exists in all populations. Disturbances could act either to increase or to decrease this level of variability (6, 7). The effect of the disturbance on population variability is dependent upon whether the disturbance acts as a deterministic or a stochastic force (9). For example, disturbance could act as a deterministic force to decrease variability by imposing a consistent selective pressure, thereby creating communities that are more similar to one another (10). Conversely, disturbance could disrupt feedback loops formed by species interactions (11) and cause initially similar communities to exhibit increased stochasticity. Under differing circumstances, both of these responses have been observed in microbial systems. For example, bacterial communities within bioreactors showed variability in composition after being disturbed with glucose additions (12). Although there were consistent functional changes in the bioreactors, there was low replicability in bacterial community composition among reactors. However, disturbances can canalize community composition under other circumstances. Roelke et al. (13) demonstrated that nutrient pulses generated predictable succession in phytoplankton communities, whereas undisturbed communities diverged along chaotic compositional trajectories. However, because experiments studying the variability of microbial communities often use different disturbances and metrics of variation, it is difficult to draw general conclusions about the effect of environmental stress on community variability.

Predicting the composition of microbial communities using environmental disturbances is a major objective of microbial ecology (14). Here, we define a disturbance as an external force that perturbs ecological communities in such a way that it selectively favors or disfavors specific populations or interferes with community processes (15). Several studies have stated that their goal was to understand how environmental disturbances change microbial community dynamics (16–19). However, this prediction is possible only if microbial responses to environmental forcing are repeatable. Thus, to predict microbial community responses, it is first necessary to understand how environmental drivers contribute to community variability (16). Therefore, the relationship between environmental disturbances and population-level variability is important to achieving applied goals, such as modeling microbial community composition.

To address the strong interest in understanding drivers of variability in microbial community composition, some studies have analyzed the range of compositions observed in bacterial communities following novel disturbances. These experiments have found consistent changes in community composition due to the disturbance (20, 21). Thus, strong environmental forcing induced a reproducible shift in bacterial community composition. However, experiments that analyze microbial population variability have been conducted in only a few systems, and results are often qualitative. Furthermore, these results are difficult to generalize because different ecological communities may show varied responses to the same environmental forcing (22, 23). For instance, resilient communities are characterized by a short recovery time (24, 25), so the effects of disturbance on highly resilient communities may be apparent for only a brief period. The response to multiple disturbances is even more difficult to predict, because there are often interactive and unexpected effects of the compounded stressors (26, 27). Recognizing these challenges, our experiments were designed specifically to analyze responses of two communities experiencing the same disturbances and to examine the effects of multiple disturbances.

In this study, we imposed several disturbance regimes upon periphyton communities in order to examine the effects of disturbances on the variability of populations within the periphyton. Our goal was to determine whether disturbances have repeatable effects on periphyton communities. After initially growing 108 periphyton communities on Plexiglas slides in a common environment, we then randomized each of these replicate communities into one of nine treatments, each corresponding to a different disturbance regime. To generate these nine treatments, we subjected periphyton communities to one of three possible conditions (water-scouring disturbance, disturbance by alteration of depth in the water column, or no disturbance) at two time points. These two disturbances were selected because they are both potential consequences of the high-wind events that occur in our study system (28). This 3 × 3 factorial design generated treatments that included different numbers of disturbances (0, 1, or 2 disturbance events) and different combinations of disturbances.

We quantified the variability of populations between communities within each treatment using the coefficient of variation (CV) of each taxon. The coefficient of variation is calculated as the standard deviation of the populations divided by the mean abundance of the populations and therefore has the advantage of accounting for variance-mean scaling (6). We transformed or detrended CVs as necessary to ensure that this metric was approximately normally distributed and was not biased by mean population size. Then, we used linear mixed models to compare the variability of taxa in undisturbed treatments to the variability of taxa that experienced disturbance. We separately analyzed the diatoms and bacteria found within the periphyton to compare the effects of the same disturbance regimes on these two different ecological communities.

## RESULTS

Periphyton colonized Plexiglas slides suspended in a shallow (maximum natural depth, ~4 m [28]) eutrophic lake over a period of 20 days. Experimental disturbances were then imposed at two time points (time 1 [T1] and time 2 [T2], corresponding to day 20 and day 25, respectively). At these two time points, communities were randomly assigned to conditions under which they were either left undisturbed (Ambient), disturbed by relocating the communities to a different depth in the water column (Depth), or disturbed with water scouring (Scoured). In the altered-depth disturbance, we suspended the Plexiglas slides at an 0.5-m depth, rather than a 3-m depth, for 5 days. In the water-scouring manipulation, we dragged the Plexiglas slides through the water column for 10 min at a rate of 20 to 25 cm/s. Both the Ambient slides and the Scoured slides were then replaced in the lake at 3-m depth for 5 days. The combination of these three conditions at the first time point and three conditions at the second time point created nine treatments: Ambient/Ambient (AA), Ambient/Depth Change (AD), Ambient/Scoured (AS), Depth Change/Ambient (DA), Depth Change/Depth Change (DD), Depth Change/Scoured (DS), Scoured/Ambient (SA), Scoured/Depth Change (SD), and Scoured/Scoured (SS).

### Diatom communities.

Diatom taxa on slides were enumerated by light microscopy to measure the abundance of each taxon within the periphyton biofilm. We used linear mixed models to analyze how the variability of taxa in the disturbed treatments compared to the variability of taxa in the undisturbed treatment, AA. The fixed effects in the model were 4 binary variables corresponding to whether the community received the depth disturbance at T1, the scouring disturbance at T1, the depth disturbance at T2, or the scouring disturbance at T2. A random effect for taxon was included, under the assumption that taxa have differing amounts of inherent population variability. The response variable in the linear mixed models was the square root CV of the taxon populations, measured in density per square centimeter. A lower CV corresponded to lower variability of a taxon between communities in the same treatment. The estimated treatment effects from the mixed model represent the mean differences in square root CVs between a given treatment and the undisturbed treatment, AA.

The treatment that had the highest mean taxon square root CV was the undisturbed treatment, AA. Thus, taxon populations in the AA treatment were the most variable of any treatment. The four treatments that received one disturbance, SA, AS, DA, and AD, all had significantly lower square root CVs than the AA treatment (Table 1). These treatment estimates and the corresponding *P* values were obtained from the linear mixed model for the diatom taxa. The scouring disturbance reduced the square root CV of the diatom communities by a mean of 0.155 at T1 (*P* = 0.0343) and by 0.158 at T2 (*P* = 0.0307). The altered-depth disturbance reduced the square root CV by 0.156 at T1 (*P* = 0.0330) and by 0.230 at T2 (*P* = 0.0016). Thus, densities of diatom taxa became more consistent as a result of experiencing one disturbance, regardless of whether the disturbance was applied at T1 or T2.

**TABLE 1:** Mixed model results for diatoms^a^

Disturbance	Estimated effect	*P* value
Intercept (AA)	1.251	NA^b^
T1: D	−0.156	0.0330*
T1: S	−0.155	0.0343*
T2: D	−0.230	0.0016**
T2: S	−0.158	0.0307*
T1: D × T2: D	0.207	0.0458*
T1: S × T2: D	0.102	0.3220
T1: D × T2: S	0.109	0.2899
T1: S × T2: S	0.217	0.0363*
Random effect		
Taxon	0.0240	NA

a Results of the linear mixed model using disturbances at T1 and T2 as predictors of the taxon-level variability (as given by the square root of the taxon CVs) of diatom communities from the nine experimental treatments. Disturbance effect estimates are given in comparison to the undisturbed treatment, AA, which is why there is no *P* value estimate for the AA treatment. Each of the single disturbances at T1 and T2 significantly reduced the average taxon square root CV. There were significant positive interactions for communities that received the same disturbance at T1 and T2, corresponding to the DD and SS treatments. No *P* value was calculated for the random effect, because we were not interested in testing how much variability was explained by differences between taxa. *, *P* < 0.05; **, *P* < 0.01.

b NA, not applicable.

In both the DD and SS treatments, there were significant positive interactions between the disturbances at T1 and T2 (*P* = 0.0458 and *P* = 0.0363, respectively). In these treatments, which received the same type of disturbance at T1 and T2, the taxa were more variable than would be expected from the independent effects of the disturbances at T1 and T2. However, there was no significant interaction between disturbances in communities that experienced different types of disturbances at T1 and T2 (corresponding to treatments DS and SD). Therefore, communities that received different disturbances at the two time points continued to become less variable as a result of experiencing another disturbance, whereas communities that experienced the same disturbance twice did not become as consistent.

We performed principal component analyses (PCAs) to determine if the community composition shifted as a result of the disturbances. We compared the undisturbed treatment, AA, to the disturbed treatments to identify whether the disturbed communities separated from the AA treatment in community composition. Strong separation of disturbed and undisturbed communities would indicate novel community development in the disturbed treatments. The PCA for the diatom communities captured 97.2% of community variability in the first two axes. The first axis, responsible for 92.8% of variability, represented the tradeoff between communities dominated by *Gomphonema* spp. and those dominated by *Nitzschia holsatica*. The loadings for these two taxa on the first axis were −0.650 and 0.757, respectively. The second eigenvector accounted for 4.4% of variability and corresponded to *Cocconeis* spp., colonial *Fragilaria*, *Gomphonema* spp., and *Nitzschia holsatica* with loadings of −0.481, −0.428, 0.601, and 0.465, respectively.

The communities in the undisturbed treatment, AA, occupied a large area of the PCA space (see Fig. 2A). Communities from the AA treatment spanned nearly the entire length of the first axis and had both the highest and lowest points on the second axis. This PCA indicates that communities within the AA treatment were highly variable, even in the context of the other, disturbed communities. Additionally, the majority of disturbed communities occur within the area spanned by the AA treatment, suggesting that there are no major differences in community composition between the AA treatment and the disturbed treatments.

### Bacterial communities.

Bacterial community composition in the periphyton was determined using a PCR-based DNA fingerprinting method called automated ribosomal intergenic spacer analysis (ARISA) (29). This method generates a measure of the relative abundance of each population that was amplified by PCR, allowing for rapid comparisons of many samples. Each detected amplicon corresponds to an operational taxonomic unit (OTU). We used a linear mixed model analysis with the same structure as the mixed model for the diatoms but using the normalized ARISA peak height as a measure of population relative abundance. Again, we used this analysis to compare the variability of taxa in communities experiencing disturbances to the variability of taxa in the undisturbed treatment, AA. The treatment effects and *P* values reported for the bacterial communities were obtained from this analysis.

**FIG 1:**
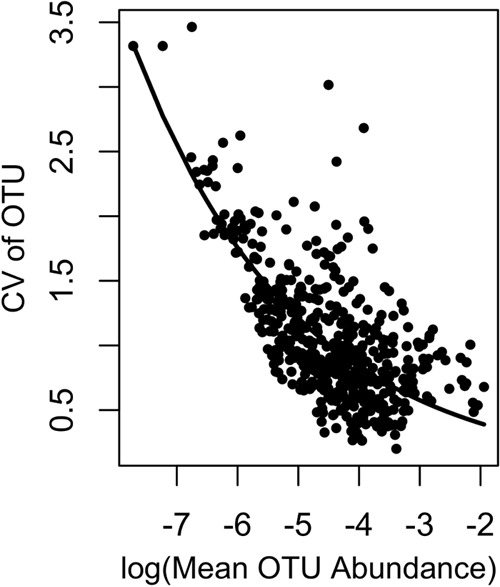
We detrended the CVs of OTUs from the ARISA data because the CVs were strongly related to mean OTU relative abundance. We expected the CVs of the OTUs to decrease as OTUs became more abundant. Thus, we fitted an exponential function to the data and used the residuals of this relationship in the subsequent mixed model.

The analyses for the bacterial communities used the residuals from detrended taxon CVs as the response variable. Detrending was performed because the CVs of the OTUs showed a strong relationship with mean OTU abundances, whereby OTUs with higher mean abundances had lower CVs. To remove the effect of mean abundance, we fitted an exponential model of the OTU CVs as a function of log(OTU mean abundances) (Fig. 1). We then used the residuals of this model as the metric of variability for each OTU, because OTUs with positive residuals were more variable than expected, whereas OTUs with negative residuals were less variable than expected. Because we used these model residuals instead of the OTU CVs, the effect sizes and standard errors are smaller in the bacterial analysis than in the diatom analysis.

For the bacterial communities, AA had the median level of variability out of the nine treatments. However, no treatment was significantly more variable than the AA treatment. No single disturbance at either T1 or T2 had significant effects on the variability of OTUs (Table 2). Thus, the OTUs in the treatments SA, AS, DA, and AD did not strongly differ in variability from those in the undisturbed treatment, AA. However, three out of the four terms for interactions between disturbances at T1 and disturbances at T2 were significant and negative; the treatments DD, SD, and SS had lower CVs than would have been predicted by the additive effects of disturbances at T1 and T2. Thus, the significant interaction terms show that the responses of the DD, SD, and SS treatments differed substantially from the independent effects of single disturbances. The effect sizes of these interactions show that DD, SD, and SS were the three least variable treatments (Table 2).

**TABLE 2:** Mixed model results for bacteria^a^

Disturbance	Estimated effect	*P* value
Intercept (AA)	0.0163	NA^b^
T1: D	−0.0450	0.3262
T1: S	0.0565	0.2159
T2: D	0.0659	0.1510
T2: S	0.0276	0.5472
T1: D × T2: D	−0.143	0.0282*
T1: S × T2: D	−0.228	<0.001***
T1: D × T2: S	0.022	0.7350
T1: S × T2: S	−0.176	0.0065**
Random effect		
OTU	0.0459	NA

a Results of the linear mixed model using disturbances at T1 and T2 as predictors of the OTU-level variability (as given by the residuals of OTU CVs) of bacterial communities from the nine experimental treatments. As in Table 1, disturbance effect estimates are given in comparison to the undisturbed treatment, AA. There were no significant effects of single disturbances on the variability of OTUs at T1 or T2. However, there were significant negative interactions between three doubly disturbed treatments, such that treatments DD, SD, and SS were less variable than would have been expected. No *P* value was calculated for the random effect, because we were not interested in testing how much variability was explained by differences between taxa. *, *P* < 0.05; **, *P* < 0.01; ***, *P* < 0.001.

b NA, not applicable.

As with the diatom communities, we compared the bacterial community composition of the AA treatment to those of the disturbed treatments using a PCA. We evaluated whether the communities in the AA treatment separated from the communities in disturbed treatments to determine if there were consistent compositional differences between the undisturbed and disturbed communities. The PCA for the bacterial communities captured 51.2% of community variability in the first two axes. The first axis accounted for 27.6% of community variability, and the second axis accounted for 23.6% of the variability. The loadings on these two axes were primarily from the most abundant OTUs across all treatments.

**FIG 2:**
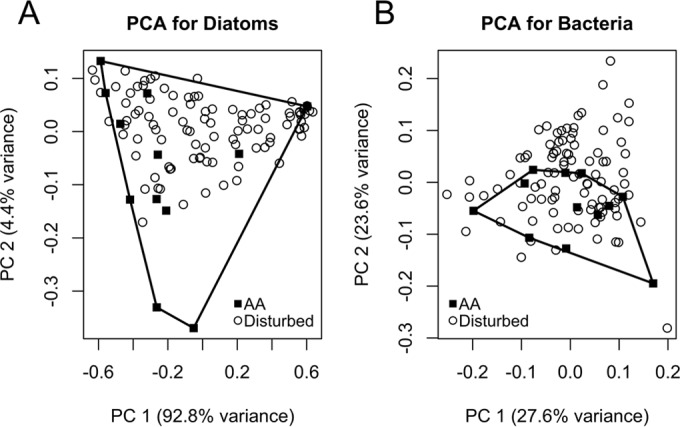
(A) Principal component analysis of the diatom communities showed that the undisturbed treatment, AA, spanned most of the space occupied by the communities in the nine treatments. The majority of disturbed communities fell within the bounds of the AA communities, showing a lack of separation between the AA treatment and the disturbed treatments. The first and second axes together account for 97.2% of community variation. The polygon depicted shows the convex hull of the AA points, which is constructed by drawing the minimum number of connections between points to encapsulate the entire set of AA points. (B) Results from the principal component analysis of the bacterial communities show that there is no strong differentiation between the community composition of the undisturbed treatment, AA, and that of the disturbed treatments. Additionally, the AA treatment covers a wide range of the PC 1 axis, which is the axis that explains the most variability between bacterial communities. The first and second axes together account for 51.2% of community variation. As above, the polygon depicted shows the convex hull of the AA points.

The communities in the undisturbed treatment, AA, occurred in close proximity to disturbed communities in PCA space (Fig. 2B). Furthermore, similarly to the diatom ordination, the AA treatment polygon overlapped with every other treatment polygon (see Fig. S5 and S6 in the supplemental material). Additionally, communities in the AA treatment fell along a wide range of the first axis, indicating that communities in this treatment showed substantial variability in community composition. Although many disturbed communities lay outside the area encompassed by the undisturbed treatment, there was no strong separation between disturbed communities and the AA communities. As with the diatom communities, these results suggest that the disturbed communities did not consistently differ from the AA communities in terms of community composition.

### Comparing the two communities.

We used three different dissimilarity metrics (Sorensen, Euclidean, and Bray-Curtis) in Mantel tests to evaluate whether differences in the diatom communities were related to differences in the bacterial communities. There was no significant relationship between the bacterial and diatom communities for any of the three metrics used (*P* = 0.540, *P* = 0.554, and *P* = 0.754 for Sorensen, Euclidean, and Bray-Curtis metrics, respectively).

**FIG 3:**
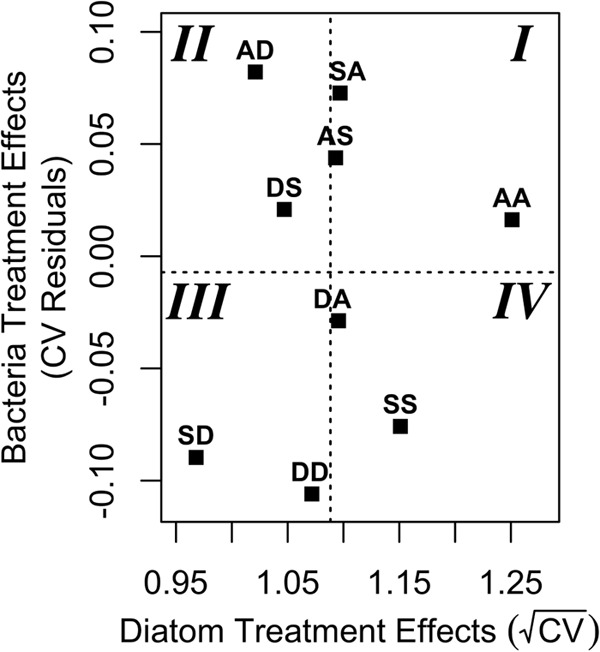
The plot shows the average variability of each treatment in the diatom and bacterial communities, as obtained from the mixed models. The dashed lines show the overall mean responses for the diatom and the bacterial treatments, such that treatments with a value higher than the mean are comparatively more variable. The AA treatment falls in the most variable portion of the plot (quadrant I), whereas the two communities that were least variable (quadrant III) were disturbed twice.

We also compared the effects of the linear mixed models of the bacteria and diatom communities. We plotted the average treatment effects from the mixed models to compare how the same treatment affected the diatom communities and the bacterial communities that cooccurred on the slides (Fig. 3). We divided the plot into four quadrants by overlaying the grand mean response of all nine treatments for the diatom and bacterial communities. Treatments with a response that was greater than the mean were relatively more variable, while treatments that fell below the mean response were relatively less variable. Treatments in quadrant I were above average in variability in both the diatom and the bacterial communities. Treatments in quadrant II were low in variability in the diatom communities but high in variability in the bacterial communities. The reverse was true of the treatments that fell in quadrant IV. Finally, treatments in quadrant III were less variable than average for both the diatom and the bacterial communities.

The AA treatment was firmly inside quadrant I, the most variable quadrant. Conversely, the only two treatments to fall within the least variable quadrant were the doubly disturbed treatments SD and DD. Furthermore, treatments experiencing the same disturbances, but in different orders, often appeared in different quadrants. The AD and DA disturbances experienced opposite effects, appearing in quadrants II and IV, respectively. Similarly, communities in the SD and DS treatments showed differing effects, particularly along the bacterial axis.

## DISCUSSION

These experiments support the hypotheses that (i) disturbances decrease the variability of populations within diatom and bacterial communities and that (ii) multiple disturbances have interactive effects. For the diatom communities, every treatment that experienced a single disturbance had a significantly lower square root CV than did the AA treatment. This consistent result shows that communities that were disturbed once became less variable than communities that were undisturbed. However, double disturbances did not necessarily cause the communities to become increasingly more consistent. Both the SS and the DD treatments had significant positive interactions, showing that these communities were more variable than would be expected based on the independent effects of the single disturbances. However, the treatments that experienced different disturbances at the two time points (SD and DS) continued to become less variable as a result of the second disturbance. Thus, for the diatom communities, different disturbances continued to increase the consistency of the diatom communities, although the same recurring disturbance appeared to be saturating in effect. Therefore, the interactions between sequential disturbances were important to understanding community dynamics in treatments that were disturbed twice.

For the bacterial communities, no single disturbance had a significant effect on the variability of OTUs within the communities. However, three of the four treatments that experienced two disturbances had interactive effects, all of which led to lower population CVs. Thus, multiple disturbances to the bacterial communities generally created communities that were less variable than communities that were disturbed once. The strong interactions indicate that multiple disturbances had novel effects on the communities, such that the communities that experienced two disturbances demonstrated much-different responses than the communities that experienced only one disturbance. Additionally, it appears that high levels of disturbance were necessary to generate changes in the bacterial communities, because the only treatments to show significant effects were disturbed twice. This was not surprising, as pelagic lake bacterial communities were previously found to be highly resilient to disturbances (25) and therefore may have recovered or experienced substantial turnover during the course of the experiment.

Despite differences in how the diatoms and the bacteria responded to individual treatments, there were several broad similarities between the diatom and bacterial responses to disturbances. For instance, none of the disturbed treatments in either the diatom or bacterial communities showed significantly greater variability than the undisturbed treatment, AA. Additionally, in both the diatom and the bacterial communities, at least two of the three least variable treatments were highly disturbed, having experienced two disturbances (Fig. 3). These results suggest that the disturbances imposed on the periphyton communities acted as canalizing ecological drivers and constrained the variability of populations within the periphyton. However, there was no overall relationship between the response of the bacterial population variability and the response of the diatom population variability (Fig. 3). In several instances, treatments were more variable than average for either the diatom or the bacterial community but less variable than average in the other community. Thus, although disturbances generally decreased variability across all communities, there was no simple relationship between changes in the bacterial communities and the diatom communities on the same slides.

The differences in the diatom and bacterial responses to individual disturbance treatments may be due to additional drivers of population variability within these two communities. For example, the strength and number of species interactions in a community can also be an important determinant of population variability (4). Although we have no estimates of species interactions in the diatom or bacterial communities, we note that the average strength and number of species interactions in these two communities may be different. This observation is based on the differing structure (richness, evenness) of the diatom and bacterial communities. Furthermore, we expect the rates of turnover to differ between the diatom and bacterial communities, with bacteria growing at a higher average rate. Thus, these various growth rates could contribute to unequal rates of turnover between the two communities, which is an important factor mediating how quickly communities recover from disturbances (25). Understanding how these various drivers of population variability interact is necessary for predicting the variability of community processes, such as changes in biomass, production, or respiration (30).

Prior work has suggested that disturbances may mediate stochastic community assembly by enforcing a niche-based environmental filter (31). Our results from the diatom communities agree with this hypothesis, as the dominant taxa in disturbed communities have traits that may confer an advantage under the disturbed conditions. For instance, some *Gomphonema* species have been found to be tolerant of turbulent conditions, showing high abundances in water currents (32). Thus, they may have been particularly resistant to the water-scouring disturbance. Similarly, *N. holsatica* is a small diatom that can become highly abundant in Icelandic lakes during the spring and summer (33). One hypothesis for the dominance of *N. holsatica* under the altered-depth disturbance is that the species reproduced rapidly under the conditions of higher light due to its small size and, therefore, high growth rate (34). Thus, we find support for the hypothesis that the harsh environmental conditions imposed by our experimental disturbances created an environmental filter, wherein taxa with functional traits favored by the disturbance could thrive under these conditions.

Although many microbial studies have demonstrated that community composition changes in response to environmental factors (e.g., references 29, 35, and 36), few have addressed the accuracy or repeatability of these results. However, studies that have evaluated the variability between disturbed microbial communities have found that there is often a high degree of similarity between strongly perturbed communities. Bell et al. (20) found that the bacterial communities following diesel contamination were similar in richness and composition following the disturbance. Similarly, Handley et al. (21) found that bacterial communities converged upon similar community compositions as a result of switching between acetate and lactate amendments. Therefore, these studies found that disturbances had repeatable effects on microbial systems, because disturbed communities were strikingly similar to one another. However, in these two cases, the communities became more consistent partially as a result of novel communities developing under altered environmental conditions. In our study, populations became less variable in the absence of novel community development; in fact, the PCA polygons for the undisturbed bacterial and the diatom communities overlap substantially with every other treatment. In this case, disturbances increased the consistency of microbial communities by placing tighter constraints on community composition.

Many studies in microbial ecology have sought to quantify the degree to which communities are shaped by stochastic versus deterministic processes (37, 38). The main deterministic process discussed is varying selection strength on microbial taxa, usually as the result of environmental or biotic stress (37, 39). Selection is named as a deterministic force under the assumption that consistent and differential selection will eventually lead to the same final community composition (40). Conversely, colonization and drift are two stochastic forces that are important in community assembly (9, 41). We find evidence in our experiments for the stochastic effects of colonization by observing the wide variability of populations in AA communities, which was presumably determined by the stochasticity of colonizers on the Plexiglas slides. Additionally, drift may be a particularly important force in communities if there is a high degree of functional redundancy (9), which can lead to communities that vary in the abundances of ecologically equivalent taxa (42). Bacterial communities, in particular, have been hypothesized to have relatively high functional redundancy of taxa due to their high species richness (43). Thus, if bacterial communities are predisposed to experience greater compositional drift due to the existence of ecologically equivalent taxa, then bacterial populations should be expected to be more variable than populations within communities with fewer ecological equivalents. This offers another explanation as to why the bacterial populations in our experiment showed no significant decrease in variability after single experimental disturbances, whereas the diatom populations did show significant decreases in variability.

Our study suggests that environmental stress can indeed act as a deterministic force in microbial communities, because the communities stressed by our disturbances became less variable after experiencing the disturbances. These results were consistent across the two experimental disturbances imposed, despite the different natures and time scales of the two disturbances. Specifically, the water-scouring disturbance was a perturbation of high impact over a short period of time (pulse disturbance), whereas the altered-depth disturbance was a sustained perturbation (press disturbance). The similar responses of the communities to these two disturbances are in line with a recent review showing that a high proportion of microbial communities are sensitive to both press and pulse disturbances; of the experiments reviewed, 92 of 112 microbial communities showed a change in composition or function in response to a pulse disturbance, and 141 of 178 communities changed in response to a press disturbance (25). Thus, the variability of microbial communities may be a useful indicator of the degree to which the communities are influenced by stochastic or deterministic processes, because many microbial communities are sensitive to disturbance. However, studying the variability of populations requires a high degree of replication, which is often lacking in microbial ecology (44). Thus, characterizing the magnitude of microbial community variability, and of the forces contributing to this variability, requires an amount of replication that is seldom found in microbial studies.

In addition to the experimental disturbances, there are many other possible factors that could have influenced the variability of populations within the periphyton. For instance, the periphyton communities experienced environmental variability throughout the duration of the experiment due to natural weather conditions and small-scale variability in environmental forces. Acknowledging this environmental stochasticity, we intentionally implemented experimental disturbances that were more extreme than the natural variability that we observed during this time period. Additionally, we did not account for colonization of diatoms or bacteria after disturbances were implemented, which may have generated additional variability in these communities. However, because the Plexiglas slides were rerandomized between disturbances, we expect systematic bias from immigration to be minimal between treatments. Furthermore, the diatom and bacterial data sets are complementary in their strengths; the diatom data were obtained through direct counts, meaning that there is high accuracy in identification, although only a subsample of the community was measured. Conversely, nearly the entire bacterial community was sampled but with some degree of bias from using ARISA (45). Thus, because the two data sets were obtained using different methods, we are confident that the similarities in the results are not an artifact of our methodology.

Prediction of microbial communities is an oft-cited goal of microbial ecology. However, predictive models can be accurate only if the process that they are describing is inherently repeatable. For instance, statistical models will produce a good fit to microbial community composition data only if these microbial communities show consistent responses to environmental drivers. The results of these experiments indicate that microbial communities do show repeatability in their response to environmental stress, because communities became more similar to one another after experiencing the same disturbance. This finding could be tested in other systems by examining whether predictive models of bacterial community composition (e.g., reference 14) have lower error rates when modeling disturbed communities. These results suggest that changes to microbial communities could be modeled using abiotic drivers as predictors. However, the diatom and bacterial communities varied in susceptibility to environmental forcing, as the effects of the same treatment on the two communities often differed. Thus, the abiotic drivers that are the best predictors of community composition are likely to vary across different ecological communities and ecosystems, as might be expected from first principles.

## MATERIALS AND METHODS

### Experimental manipulations.

These experiments were performed in Lake Myvatn, a shallow, eutrophic (external loading of 1.4 g ⋅ m^−2^ ⋅ year^−1^ of N and 1.5 g ⋅ m^−2^ ⋅ year^−1^ of P; net algal production of 222 g ⋅ m^−2^ ⋅ year^−1^ of C [46]) lake in northeast Iceland (65°40′N, 17°00′W [28]). We allowed periphyton to colonize the Plexiglas substrate for 20 days before beginning the disturbance manipulations. During this period of colonization, 108 Plexiglas slides (6 cm by 8 cm) were suspended in Lake Myvatn at a 3-m depth, which was approximately 0.3 m from the sediment surface.

Disturbances were implemented at two time points. At the first time point, day 20, we randomly assigned 36 of the 108 slides to each of the following conditions: ambient (no disturbance), altered-depth disturbance (relocation to an 0.5-m depth), or water-scouring disturbance. Weekly water column profiles showed that Secchi depth during the summer of 2013 varied between 1.5 m and 3.3 m, with 11 to 33% of surface photosynthetically active radiation (PAR) reaching 3-m depth. We chose these two disturbances (altered depth and water scouring) because they mimic natural disturbances to periphyton communities in the lake due to the high-wind events that are common at our study site (28, 47). During these high-wind events, periphyton communities may experience a change in depth due to resuspension in the water column, or individuals might be scoured from biofilms due to fast water currents.

Similarly, on day 25, we again randomized the slides into three groups and manipulated the slides with the disturbances described above, incubating the slides for another 5 days. On day 30 of the experiment, we retrieved the Plexiglas slides from the lake and froze the slides at −20°C until further processing. Additional details about the experimental manipulations are provided in Fig. S1 in the supplemental material.

10.1128/mSystems.00013-16.3 Figure S1  At the start of the experiment, the 108 Plexiglas slides were distributed across 6 identical metal racks. All 6 racks were deployed side by side on buoy lines in Lake Myvatn and were suspended 0.3 m from the sediment surface. After 20 days of periphyton colonization (T1), slides were again randomized before experiencing the first disturbance. Again, on day 25, slides were randomized onto new racks corresponding to the disturbance experienced at T2. Download Figure S1, PDF file, 0.1 MB. Copyright © 2016 Herren et al. 2016 Herren et al. This content is distributed under the terms of the Creative Commons Attribution 4.0 International license.

### Community composition analysis.

Slides were removed briefly from the freezer to obtain diatom counts on a microscope before being frozen again. Diatoms were identified to the lowest taxonomic resolution possible, which was genus or species. A minimum of 500 individuals per slide were identified by counting half-transects across slides. The mean number of individuals identified per sample was 1,063, for a total of 114,843 individuals across the 108 Plexiglas slides. We then transported the slides to Madison, WI, USA, for analysis of the bacterial communities in the periphyton using automated ribosomal intergenic spacer analysis (ARISA) (48, 49). Briefly, DNA was extracted from periphyton biomass that was scraped from the slides, and this DNA was used as the template for PCR to amplify the intergenic region between the 16S and 23S rRNA genes in bacteria. Amplicons were separated by capillary electrophoresis and used to define operational taxonomic units (OTUs). Additional details about community composition analysis are provided in the supplemental material.

### Statistical methods. (i) Diatom communities.

Because some taxa were rare and therefore were inconsistently present in samples, we analyzed only the 8 most common diatom taxa (*Nitzschia holsatica*, *Cymbella* spp., *Synedra* spp., *Gomphonema* spp., *Rhoicosphenia* spp., *Cocconeis* spp., colonial *Fragilaria* spp., and singular *Fragilaria* spp.). Together, these 8 taxa accounted for 99.4% of all individuals counted. We standardized all data to densities of each taxon per square centimeter. For each of the nine treatments, we calculated the coefficient of variation (CV) for each of the 8 taxa across the 12 slides in that treatment. We chose the CV as the indicator of population variability because it did not change in response to the mean abundance of the taxa and because it showed homogeneity of variance between treatments. Additionally, the CV integrates across all 12 slides within a treatment and mitigates the effects of any single anomalous communities. We transformed the CVs by taking their square root because the distribution of CVs was slightly skewed toward larger values.

We analyzed square root CVs using a linear mixed effects model. The four predictor variables (*X*_*D*1_, *X*_*S*1_, *X*_*D*2_, and *X*_*S*2_) were binary vectors corresponding to whether or not the taxon was in a treatment that received the depth disturbance at T1, the scouring disturbance at T1, the depth disturbance at T2, or the scouring disturbance at T2 (equation 1). We also included all interactions between these four predictor variables to assess the interactive effects of multiple disturbances. We recognized that the taxa may have different inherent levels of population variability, and so we included a random intercept by taxon, denoted by α_taxon_. This term assumes that the square root CVs of taxa are normally distributed but estimates only the distribution from which the square root CVs are drawn, rather than an effect for each taxon.


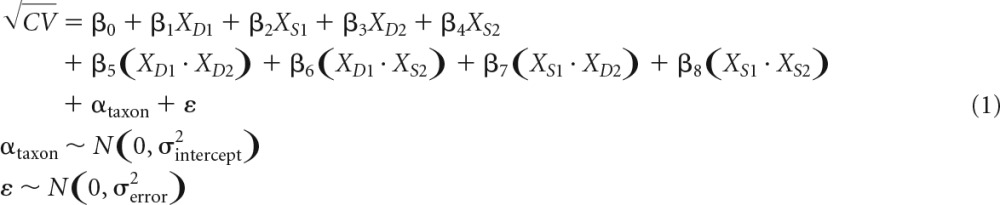


### (ii) Bacterial communities.

We removed two bacterial samples from our analyses due to their anomalously low diversities, resulting in 106 bacterial samples. When evaluating population variability, we analyzed OTUs that were present in at least 30 samples, which included 55 OTUs. For each of the 9 treatments, we calculated the CV for each OTU within that treatment. However, the CVs of the OTUs were correlated with mean OTU abundance, with highly abundant OTUs generally having lower CVs. This is a common pattern when data are relativized, due to the heteroscedasticity of binomial data (50). To account for this expected pattern, we detrended the data by fitting the CVs as a negative exponential function of the log(OTU mean relative abundance) (Fig. 1). We then used the residuals of this function as the response variable in our analyses. Points above the fitted relationship (positive residuals) are OTUs that were more variable than would be expected, after the effect of abundance was removed, whereas points below the line (negative residuals) were less variable than would be expected. We then analyzed the residuals using a statistical model analogous to that used with the diatom data (equation 2):

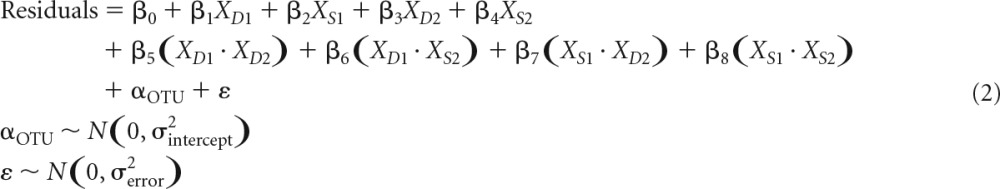



Prior to fitting the statistical model, we removed the 6 outliers that were greater than 3 standard deviations from the mean of the residuals. However, the model was robust to these outliers and identified the same treatments as significant when the outliers were included. The results were also robust to changes in the frequency cutoff used to determine the number of OTUs included; the model identified the same treatments as significant when varying the cutoff for inclusion in the analysis between presence in at least 20 samples and presence in at least 40 samples. Additional information on statistical methods and diagnostics can be found in the supplemental material (see Fig. S2 to S4).

10.1128/mSystems.00013-16.4 Figure S2  We plotted the CVs of each diatom taxon within each treatment (72 total populations) against the mean abundance of that population. We found that the population CV was not biased by the mean population abundance. Download Figure S2, PDF file, 0.3 MB. Copyright © 2016 Herren et al. 2016 Herren et al. This content is distributed under the terms of the Creative Commons Attribution 4.0 International license.

10.1128/mSystems.00013-16.5 Figure S3  We plotted the estimated random effect from the mixed model against the log of the mean abundance of each diatom taxon. We found no relationship between the fitted random effects and the mean abundances of the diatom taxa. Abbreviations refer to *Cocconeis* spp., *Rhoicosphenia* spp., *Cymbella* spp., *Synedra* spp., *Gomphonema* spp., *Nitzschia holsatica*, single *Fragilaria* spp., and colonial *Fragilaria* spp. Download Figure S3, PDF file, 0.3 MB. Copyright © 2016 Herren et al. 2016 Herren et al. This content is distributed under the terms of the Creative Commons Attribution 4.0 International license.

10.1128/mSystems.00013-16.6 Figure S4  Mixed model residuals were not biased by treatment and were approximately normally distributed. Download Figure S4, PDF file, 0.3 MB. Copyright © 2016 Herren et al. 2016 Herren et al. This content is distributed under the terms of the Creative Commons Attribution 4.0 International license.

10.1128/mSystems.00013-16.7 Figure S5  A principal component analysis for the diatom communities shows that the AA treatment polygon overlaps strongly with every other treatment polygon. The AA treatment polygon (red) spans a large portion of the first axis (PC 1), as well as the entire length of the second axis (PC 2). Download Figure S5, PDF file, 0.4 MB. Copyright © 2016 Herren et al. 2016 Herren et al. This content is distributed under the terms of the Creative Commons Attribution 4.0 International license.

10.1128/mSystems.00013-16.8 Figure S6  A principal component analysis for the bacterial communities shows that the AA treatment polygon (red) overlaps strongly with every other treatment polygon. Download Figure S6, PDF file, 0.5 MB. Copyright © 2016 Herren et al. 2016 Herren et al. This content is distributed under the terms of the Creative Commons Attribution 4.0 International license.

### (iii) PCAs of communities.

We performed principal component analyses (PCAs) on the diatom and the bacterial communities. Our main goal for these analyses was to evaluate whether the composition of the disturbed communities consistently differed from the composition of undisturbed communities. Because there was a wide range in the mean densities of diatoms on the slides, we transformed the diatom counts into relative abundances before running this analysis. Again, we used only the 8 most common taxa in the diatom PCA. Similarly, in the PCA of the bacterial communities, we removed all OTUs present in fewer than 30 samples.

### (iv) Comparing the two communities.

To assess whether there were correlations between the diatom communities and the bacterial communities, we performed Mantel tests on the 106 slides for which we had data on both the diatom and bacterial communities. We used these tests to determine if changes to either the diatom or the bacterial community on a slide could predict changes in the other community.

10.1128/mSystems.00013-16.1 Text S1  We provide additional details about the design and data collection of our experiment. We performed diagnostics on the mixed models used to statistically analyze our results so as to verify the appropriateness of these models. Download Text S1, DOCX file, 0.1 MB. Copyright © 2016 Herren et al. 2016 Herren et al. This content is distributed under the terms of the Creative Commons Attribution 4.0 International license.

10.1128/mSystems.00013-16.2 Text S2  Approximations for community proportion CVs. We performed simulations to demonstrate that the pattern seen in the CVs of the bacterial data set is an expected result of relativizing the data. Download Text S2, PDF file, 0.1 MB. Copyright © 2016 Herren et al. 2016 Herren et al. This content is distributed under the terms of the Creative Commons Attribution 4.0 International license.

10.1128/mSystems.00013-16.9 Table S1  Definitions of community properties and their relationships to population variability. Download Table S1, DOCX file, 0.1 MB. Copyright © 2016 Herren et al. 2016 Herren et al. This content is distributed under the terms of the Creative Commons Attribution 4.0 International license.

10.1128/mSystems.00013-16.10 Table S2  For each treatment, we calculated a pseudo-*P* value, which was the fraction of the null distribution of mixed model coefficients that was lower than the observed mixed model coefficient. *, *P* < 0.05; **, *P* < 0.01. Download Table S2, DOCX file, 0.1 MB. Copyright © 2016 Herren et al. 2016 Herren et al. This content is distributed under the terms of the Creative Commons Attribution 4.0 International license.
