# Supplementary figures and images for: Environmental Disturbances Decrease the Variability of Microbial Populations within Periphyton
Source: mSystems. 2016 May 17;1(3):e00013-16. doi: 10.1128/mSystems.00013-16 (PMC5072133; doi:10.1128/mSystems.00013-16)

| T0 (Day 1)                                                                          |                                                                                     | T1 (Day 20)          |                                                                                     | T2 (Day 25)                                                                         |                      |                                                                                       |                                                                                       |
|-------------------------------------------------------------------------------------|-------------------------------------------------------------------------------------|----------------------|-------------------------------------------------------------------------------------|-------------------------------------------------------------------------------------|----------------------|---------------------------------------------------------------------------------------|---------------------------------------------------------------------------------------|
| 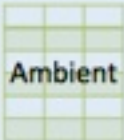   | 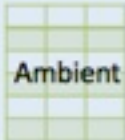   | <b>Randomization</b> | 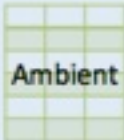   | 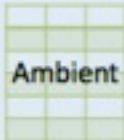   | <b>Randomization</b> | 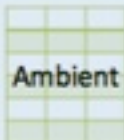   | 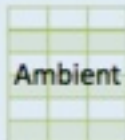   |
| 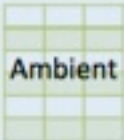 | 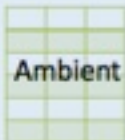 |                      | 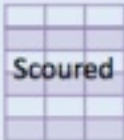 | 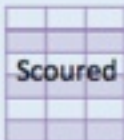 |                      | 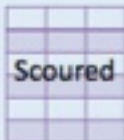 | 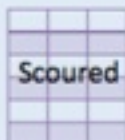 |
| 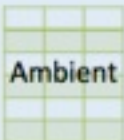 | 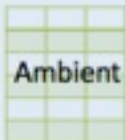 |                      | 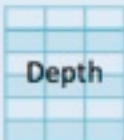 | 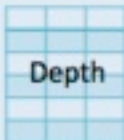 |                      | 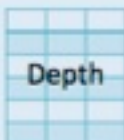 | 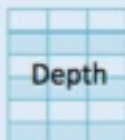 |

Supplement: Figure S1 [file sys003162022sf4.pdf]

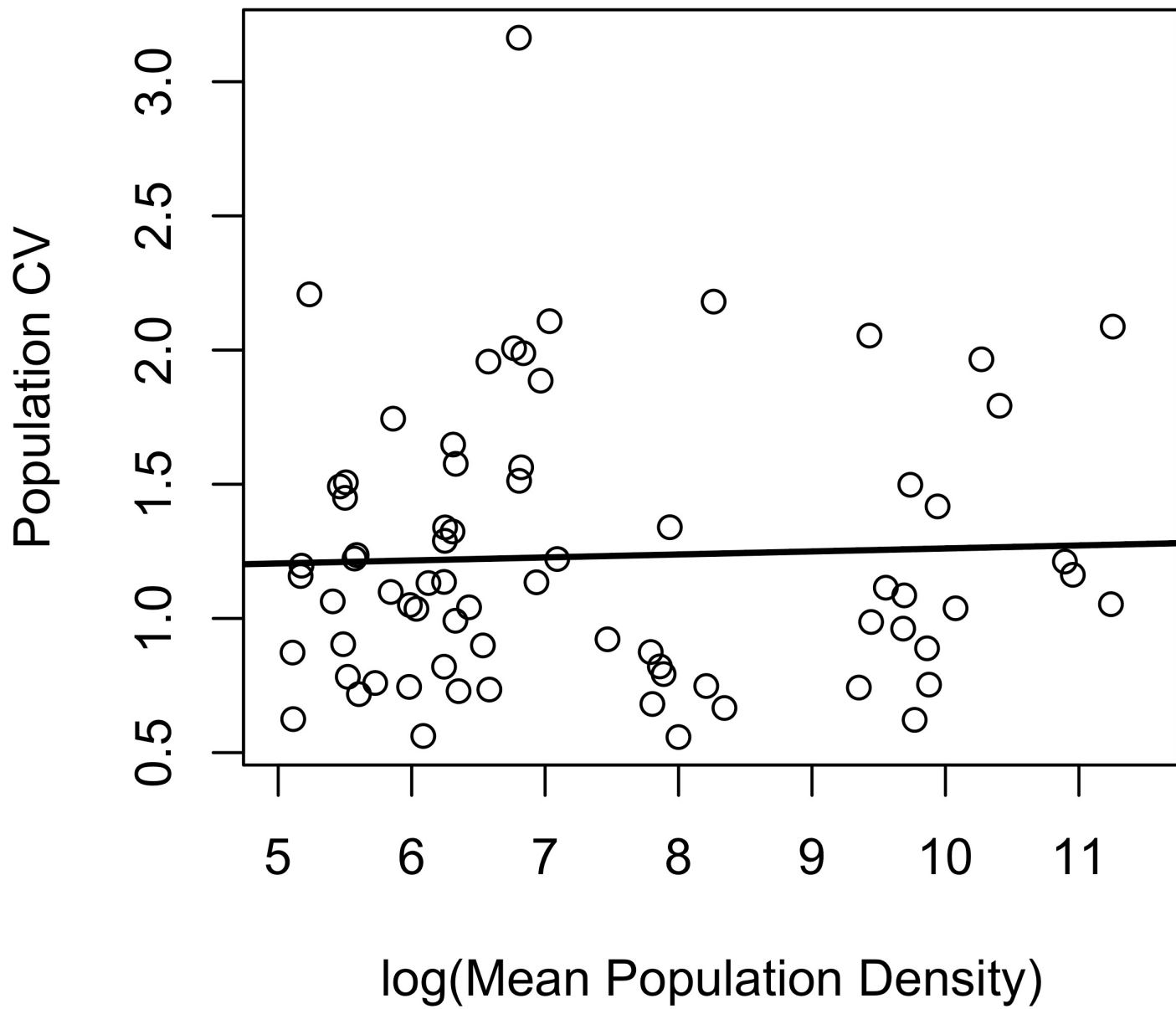

Supplement: Figure S2 [file sys003162022sf5.pdf]

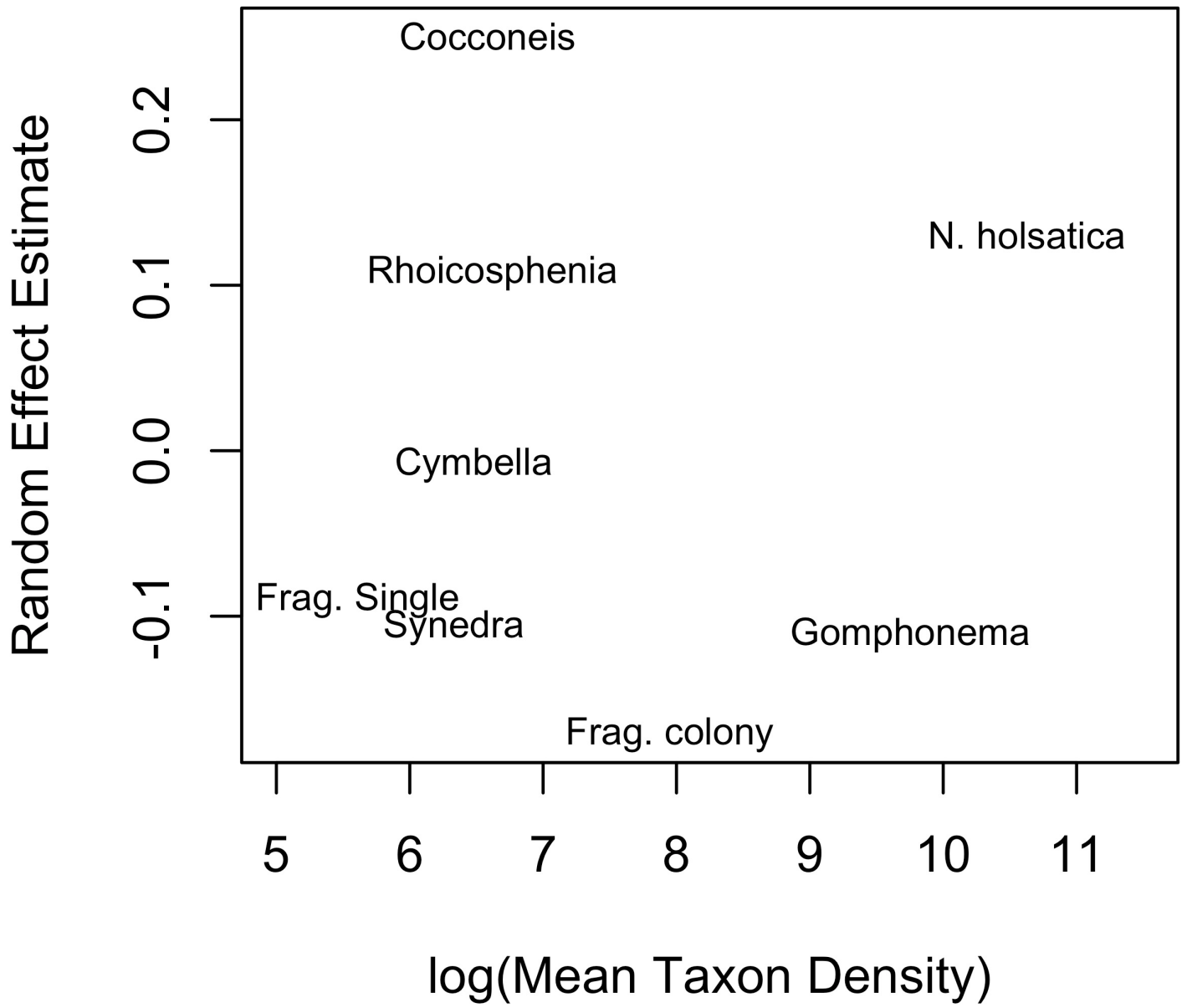

Supplement: Figure S3 [file sys003162022sf6.pdf]

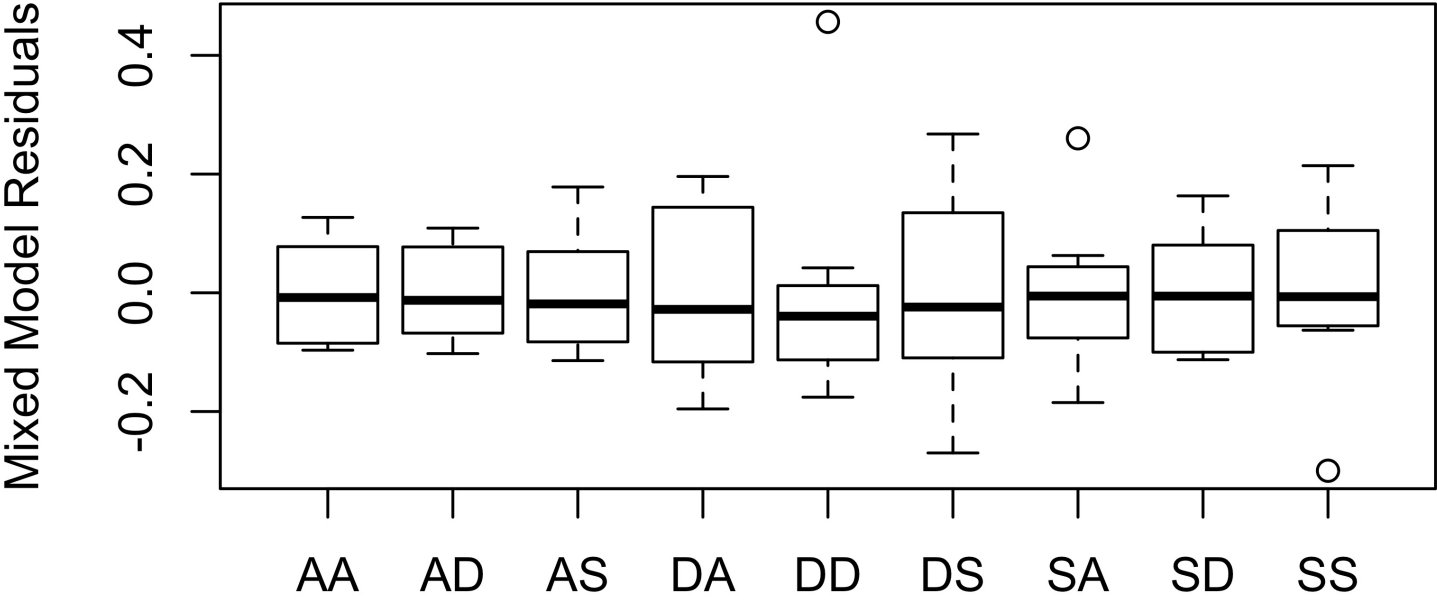

Supplement: Figure S4 [file sys003162022sf7.pdf]

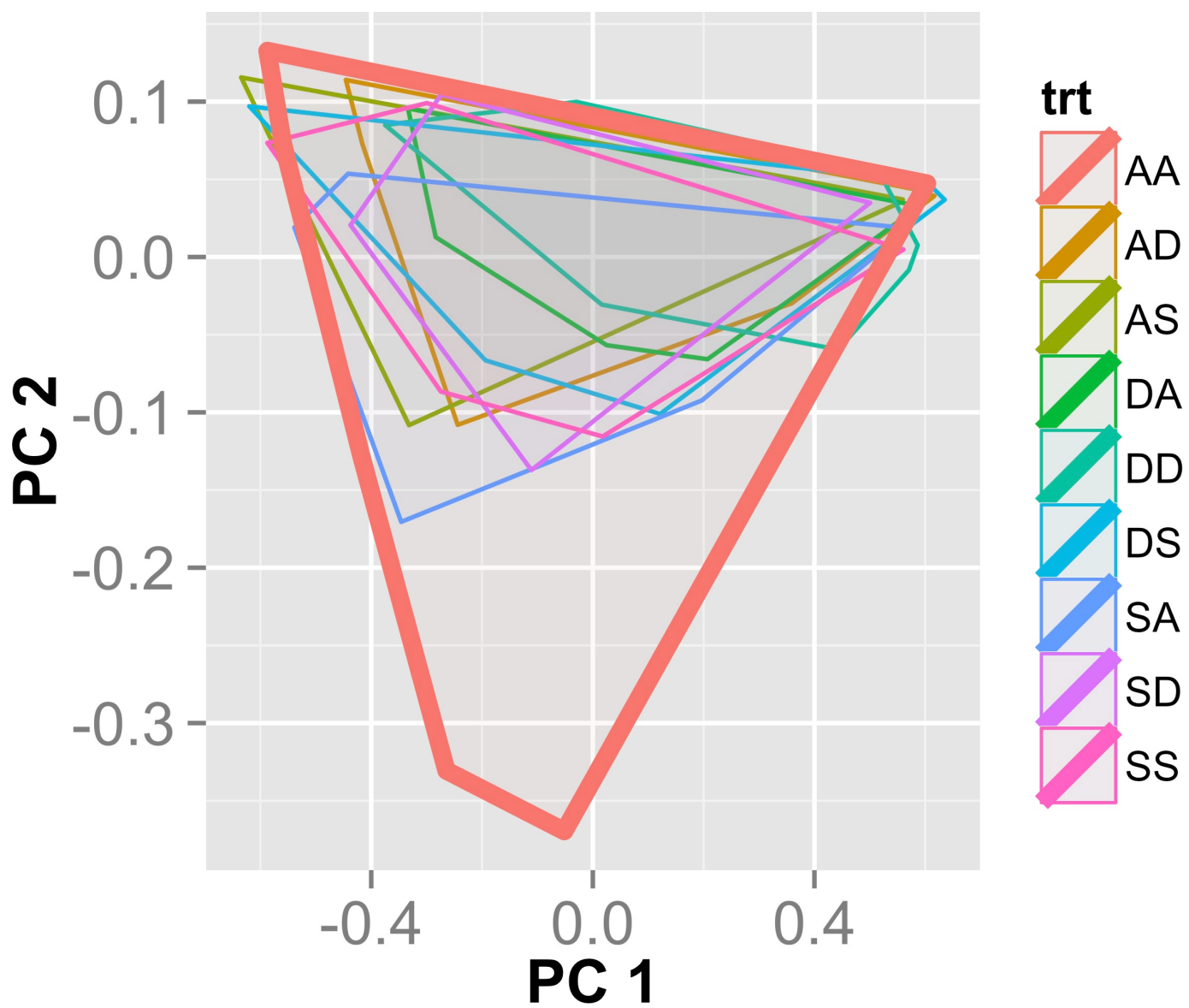

Supplement: Figure S5 [file sys003162022sf8.pdf]

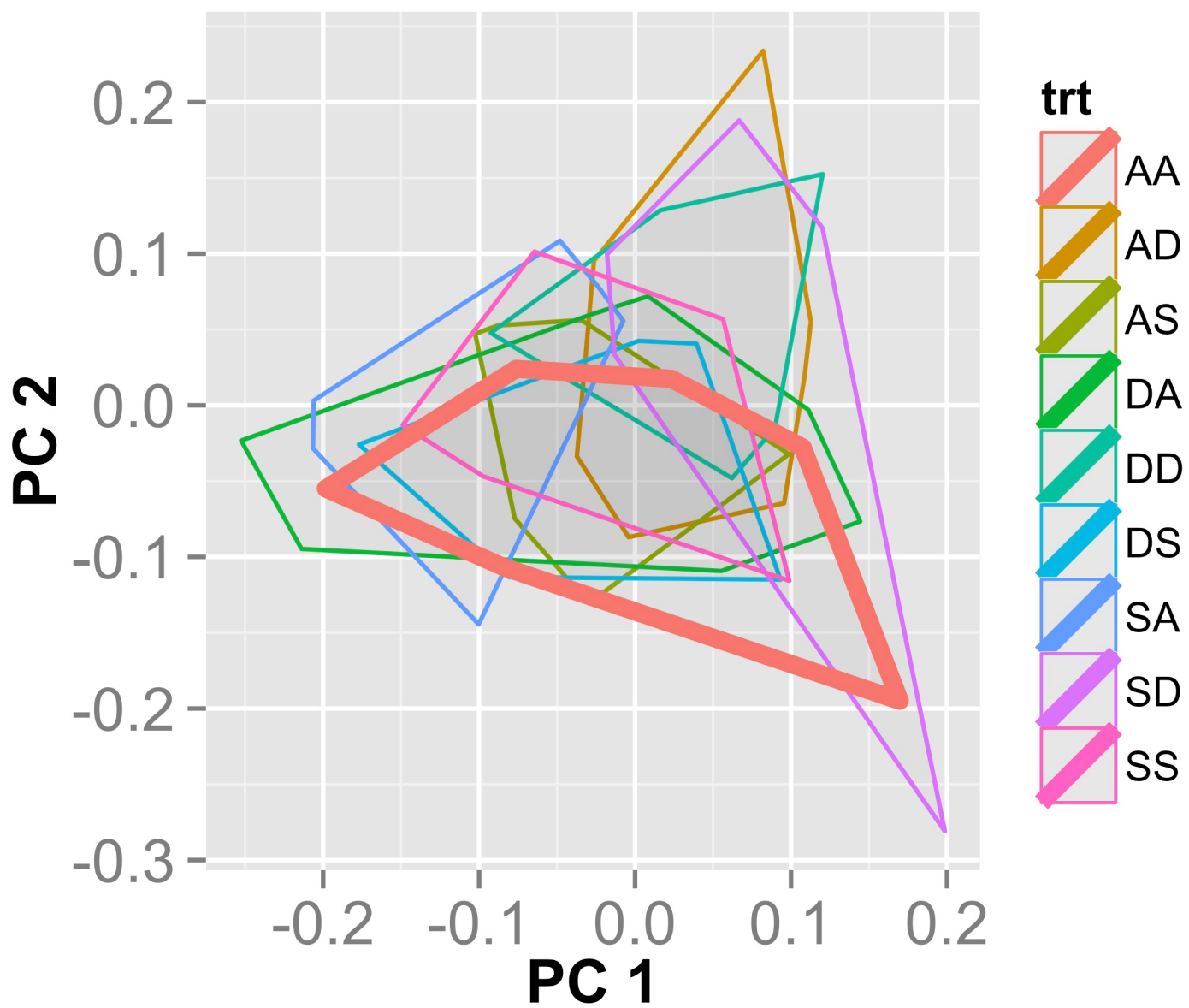

Supplement: Figure S6 [file sys003162022sf9.pdf]
