# Supplementary material for: Environmental Disturbances Decrease the Variability of Microbial Populations within Periphyton
Source: mSystems. 2016 May 17;1(3):e00013-16. doi: 10.1128/mSystems.00013-16 (PMC5072133; doi:10.1128/mSystems.00013-16)
Supplement: Text S1 [file sys003162022s1.docx]

Supplementary Online Materials and Methods

**Title:** Environmental disturbances decrease the variability of microbial communities within periphyton

**Authors:** Cristina M. Herren^1^, Kyle C. Webert^2^, and Katherine D. McMahon^3^

**Author affiliations:**

**^1^**Freshwater and Marine Sciences Program, University of Wisconsin - Madison, Madison, Wisconsin, USA

cherren@wisc.edu

^2^Department of Zoology, University of Wisconsin - Madison, Madison, Wisconsin, USA

^3^Departments of Bacteriology and Civil and Environmental Engineering, University of Wisconsin - Madison, Madison, Wisconsin, USA

**Additional Materials and Methods**

*Experimental Manipulations*

Periphyton was chosen as a study system because of the high species diversity of benthic algae in periphyton and the ability to analyze the co-occurring algal and bacterial communities. Additionally, diatoms at Lake Myvatn are particularly important to the lake food web. Lake Myvatn is a shallow, eutrophic lake that has high diatom production due to elevated concentrations of nutrients in the lake’s groundwater inputs (Einarsson et al 2004).

Plexiglas slides (6cm x 8cm) were used as the substrate for periphyton growth. Two holes were drilled in the Plexiglas in order to attach slides to metal racks. Twelve replicates of each of the 9 treatments (108 total slides) were uniquely labeled and distributed randomly among six metal racks (18 slides per rack). Slides were randomized before the start of the experiment and before each of the two disturbances (Fig. S1). The bottom of the rack was attached to an anchor that sank into the lake sediment, and a line with a buoy was attached to the top of the rack to suspend the rack vertically. On this top line, there was also a smaller stabilization buoy secured 0.5m above the rack. All racks were deployed within a 5m radius.

We took care to minimally perturb the periphyton communities aside from the two disturbance treatments described. During disturbance manipulations, we retrieved all 6 racks and placed the slides on new racks corresponding to the treatment they were designated to receive at the next time point. In the time between removal from the initial rack and attachment to the next rack, each slide was stored individually in a covered container filled with lake water. The 36 slides receiving no disturbance were distributed across 2 racks and were simply replaced back into the water column at 3m depth. The 36 slides receiving the water scouring disturbance were distributed across 2 racks that were dragged through the water at 20-25cm/s for 10 minutes, simulating a strong current in the lake due to a high wind event. After this disturbance, these racks were also replaced into the lake at a depth of 3m. The last set of 36 slides, which received the altered depth disturbance, were distributed across 2 racks and were replaced in the water column at a depth of 0.5m. These two racks remained at this shallower depth for 5 days, until the time of the second disturbance.

*Diatom Counts*

Slide counts were performed with a Leica compound microscope at 400x magnification. The vast majority (>99%) of algae on the slides were diatoms. In order to account for potential effects of spatial heterogeneity within each slide, half transects were counted across the Plexiglas slide, and only the center 6cm x 6cm area was counted. Diatoms were identified to the lowest taxonomic resolution possible, which was genus or species. Half transects were counted completely until a minimum of 500 individuals were identified. The mean number of individuals counted per sample was 1063, with 114,843 total individuals counted in the 108 samples. Seventeen taxonomic groupings were differentiated, and fewer than 1% of cells were not identifiable. Slides were again frozen after counting.

*Bacteria Analysis Using ARISA*

Slides were transported back to Madison, Wisconsin, USA, for analysis of the bacterial communities in the periphyton using Automated Ribosomal Intergenic Spacer Analysis (ARISA). The same 6cm x 6cm area of the slide that was counted for periphyton was scraped with a sterile razor blade and transferred to a microcentrifuge tube. DNA from these samples was extracted using a xanthogenate-phenol-chloroform protocol described elsewhere by Miller and McMahon (2011). We then used PCR with universal bacterial primers 1406f (5’-TGYACACACCGCCCGT-3’) and 23Sr (5’-GGGTTBCCCCATTCRG-3’) to amplify the intergenic spacer region between 16S and 23S of the bacteria in these samples. PCR reactions used 5uL of 10x buffer, 2uL MgCl2, 1.25uL of dNTPs, 1uL of each primer, 1uL of template DNA, 0.25uL of DNA polymerase, and 13.5uL of water. Samples were analyzed using denaturing capillary electrophoresis using an ABI 3730 at the University of Wisconsin Biotechnology Center. ARISA output was calibrated against a 100-2000bp standard (Bioventures) and was analyzed using the GeneMarker v 1.5 software (SoftGenetics LLC) and custom R scripts (Jones and McMahon 2009). The output of this software resulted in relative abundance tables of the operational taxonomic units (OTUs) present in the samples.

**Additional Statistical Methods and Diagnostics**

*Diagnostics for Mixed Models*

In order to validate that the mixed model approach was appropriate for these data, we ran several diagnostic tests on the diatom data. First, we ensured that the measure of variability (the taxon CV) was not biased by mean taxon abundance. Using a linear regression, we found no effect of mean abundance on the CV of these populations (t = .34, p = 0.74, Fig. S2).

Similarly, we plotted the random effects estimated in the mixed model to identify whether these fitted effects were biased by the mean abundances of the taxa. We found no relationship between the estimated random effects and the average log mean abundance of the diatoms (Fig. S3). Thus, rare taxa were not more variable than common taxa, suggesting that sample sizes of the taxa were sufficiently large that the variability of taxa was not substantially influenced by sampling error.

Additionally, we checked that the residuals from the mixed model were not biased by treatment and were approximately normally distributed (Fig. S4).

*Additional Statistical Methods and Validation*

In order to assess whether the results of our analyses were robust to statistical methodology, we validated our results using a secondary analysis. Here, we present the results of this supplementary analysis conducted on the diatom data. This test gave similar results to the mixed model shown in the main text, as it also showed that the disturbed treatments were significantly lower in variability than the control treatment, AA.

In this second analysis, we bootstrap a mixed model similar to the one presented in the main text in order to obtain empirical p values for treatment effects, rather than relying upon p values obtained directly from the mixed model. We used this approach to verify that the significant treatment effects were not the result of a violation of the assumptions of a mixed model. Specifically, we were concerned that using taxon CVs as the statistical unit may inflate the probability of finding spurious statistical significance; the taxa were all part of the same communities on the Plexiglas slides, which could lead to interdependence among taxon CVs. Thus, to address this concern, we used a bootstrapping approach to account for any effect the co-occurrence of taxa on the same slide might have on the estimated treatment effects. We used this null model obtained from the bootstrap to discern significance, rather than relying upon the theoretical distribution of treatment effects.

To validate our statistical methods, we compared the p values for treatment effects that were obtained from the two methodologies described above. For ease of comparison, the model that we used for this validation was the simplest possible mixed model for our diatom data; we analyzed the square root CVs of the diatom taxa as a function of the 9 disturbance treatments with a random effect for taxon (Eq. S1). This model structure avoids the use of interaction terms. Thus, the treatment effects from this model give the estimated differences between of the square root CVs of the ambient treatment, AA, and each of the 8 disturbed treatments.

$\begin{matrix} \sqrt{CV}=\beta_{ 0} +\beta_{ 1} X_{AD}+\beta_{ 2} X_{AS}+\beta_{ 3} X_{DA}+\beta_{ 4} X_{SA} \\ +\beta_{ 5} X_{DD}+\beta_{ 6 } X_{DS}+\beta_{ 7} X_{SD}+\beta_{ 8} X_{SS} \\ + \alpha_{taxon}+ \varepsilon\\ \alpha_{taxon}\sim N(0 , \sigma_{intercept}^{2}) \\ \varepsilon\sim N(0 , \sigma_{error}^{2}) \end{matrix}$ (Eq. S1)

*P values obtained from the theoretical distribution*

As in the main text, the p values obtained directly from the mixed model are the result of calculating the z score of the observed treatment effect in order to find the proportion of theoretical treatment effects that were more extreme than the observed treatment effect.

*P values obtained using bootstrapping*

In the bootstrapping approach, we obtained an empirical null distribution of treatment effects by randomizing the data before running the mixed model. We randomly assigned the 108 slides to the 9 treatments before calculating the square root CVs of the diatom taxa. In this approach, taxa remain associated by slide, but there is no true effect of treatment, because slides are randomly assigned to treatments. Then, we ran the mixed model on these square root CVs obtained from the randomized data and recorded the treatment estimates. Thus, the treatment estimates should include any effects of the interdependence of the taxa on the same slide. We repeated this workflow 1000 times to obtain a distribution of 1000 sets of treatment estimates. These 1000 sets of treatment estimates were then used as the null distribution of treatment effects, as they were all obtained under the condition where there was no real difference between treatments. Finally, we compared the treatment effects from the real, non-randomized dataset to the treatment effects obtained when slides were randomized into treatments. We obtained pseudo p values by calculating the proportion of treatment estimates in the null distribution that were more extreme (lower) than treatment estimates from the real dataset. This proportion gives the fraction of random slide assignments that led to a more negative estimated treatment effect than was observed in the true data.

We found that, similar to the mixed model, the overall trend was for populations to become more predictable (have a lower square root CV) after the experimental disturbances. The associated pseudo p values are given in Table S1. The similarity of the results of this analysis and the analysis presented in the main text suggests that the observed decrease in population variability in disturbed treatments is a result that is robust to various statistical methodologies.

**Fig. S1:** At the start of the experiment, the 108 Plexiglas slides were distributed across 6 identical metal racks. All 6 racks were deployed side by side on buoy lines in Lake Myvatn and were suspended 0.3m from the sediment surface. After 20 days of periphyton colonization (T1), slides were again randomized before experiencing the first disturbance. Again, on day 25, slides were randomized onto new racks corresponding to the disturbance experienced at T2.

**Fig. S2**: We plotted the CVs of each diatom taxon within each treatment (72 total populations) against the mean abundance of that population. We found that the population CV was not biased by the mean population abundance.

**Fig. S3**: We plotted the estimated random effect from the mixed model against the log of the mean abundance of each diatom taxon. We found no relationship between the fitted random effects and the mean abundances of the diatom taxa. Abbreviations refer to *Cocconeis spp., Rhoicoshpenia spp., Cymbella spp., Synedra spp., Gomphonema spp., Nitzschia holsatica,* single *Fragilaria spp.*, and colonial *Fragilaria spp.*

**Fig. S4**: Mixed model residuals were not biased by treatment and were approximately normally distributed.

**Fig. S5:** A principal component analysis for the diatom communities shows that the AA treatment polygon overlaps strongly with every other treatment polygon. The AA treatment polygon (red) spans a large portion of the first axis (PC 1), as well as the entire length of the second axis (PC 2).

**Fig. S6:** A principal component analysis for the bacterial communities shows that the AA treatment polygon (red) overlaps strongly with every other treatment polygon.

**Supplementary Materials Works Cited**

**Colwell RK**. 1974. Predictability, Constancy, and Contingency of Periodic Phenomena. Ecology **55**:1148–1153.

**Pimm SL**. 1984. The complexity and stability of ecosystems. Nature **307.5949**: 321-326.

**Einarsson Á**, **Stefánsdóttir G**, **Jóhannesson H**, **Ólafsson JS**, **Gíslason GM**, **Wakana I**, **Gudbergsson G**, **Gardarsson A**. 2004. The ecology of Lake Myvatn and the River Laxá: Variation in space and time. Aquatic Ecology **38**:317–348.

**Miller TR, McMahon KD**. 2011. Genetic diversity of cyanobacteria in four eutrophic lakes."FEMS microbiology ecology **78.2**: 336-348.

**Jones SE, McMahon KD**. 2009. Species‐sorting may explain an apparent minimal effect of immigration on freshwater bacterial community dynamics. Environmental microbiology **11.4**: 905-913.
