# Supplementary material for: Environmental Disturbances Decrease the Variability of Microbial Populations within Periphyton
Source: mSystems. 2016 May 17;1(3):e00013-16. doi: 10.1128/mSystems.00013-16 (PMC5072133; doi:10.1128/mSystems.00013-16)
Supplement: Text S2 [file sys003162022s10.pdf]

Approximations for Community Proportion CVs

Here, we simulate data to demonstrate the transformation of coefficients of variation (CVs) when data are relativized. We begin by assuming that populations are approximately Poisson-distributed. This assumption also generates total community abundances (sums of all taxa in communities) that are Poisson-distributed.

```
#Make CV function
cv <- function(vec){
  eval.cv <- sd(vec) / mean(vec)
  return(eval.cv)
}
```

If X and Y are Poisson-distributed variables, X / (X + Y) can be approximated by Beta(X, Y), so long as X and Y are sufficiently large. We use this fact to show that relativizing Poisson data approximates a binomial process. We define our binomial composition process by assuming that each the number of taxa in each community is the same, given by X + Y, and that the number of individuals from population X is binomially distributed with a probability X / (X + Y). At sufficiently large X and Y, the proportion of individuals X in the overall population also converges upon the distribution Beta(X, Y) * K, where K is a normalizing constant. We show this with simulations:

```
num.samp <- 10000
X <- 200
Y <- 3000
par(mfrow = c(3, 1))
p1 <- rpois(num.samp, X)
p2 <- rpois(num.samp, Y)
hist(p1/(p1 +p2) , xlim = c(.04, .08), main = "Poisson
Model")
b1 <- rbeta(num.samp, X, Y)
hist(b1, xlim = c(.04, .08), main = "Beta Distribution")

#Compare the above distribution to data generated from
binomial distribution
trials <- X + Y
bn1 <- rbinom(num.samp, trials, X / (X + Y))
hist(bn1 / trials, xlim = c(.04, .08), main = "Binomial
Model")
```

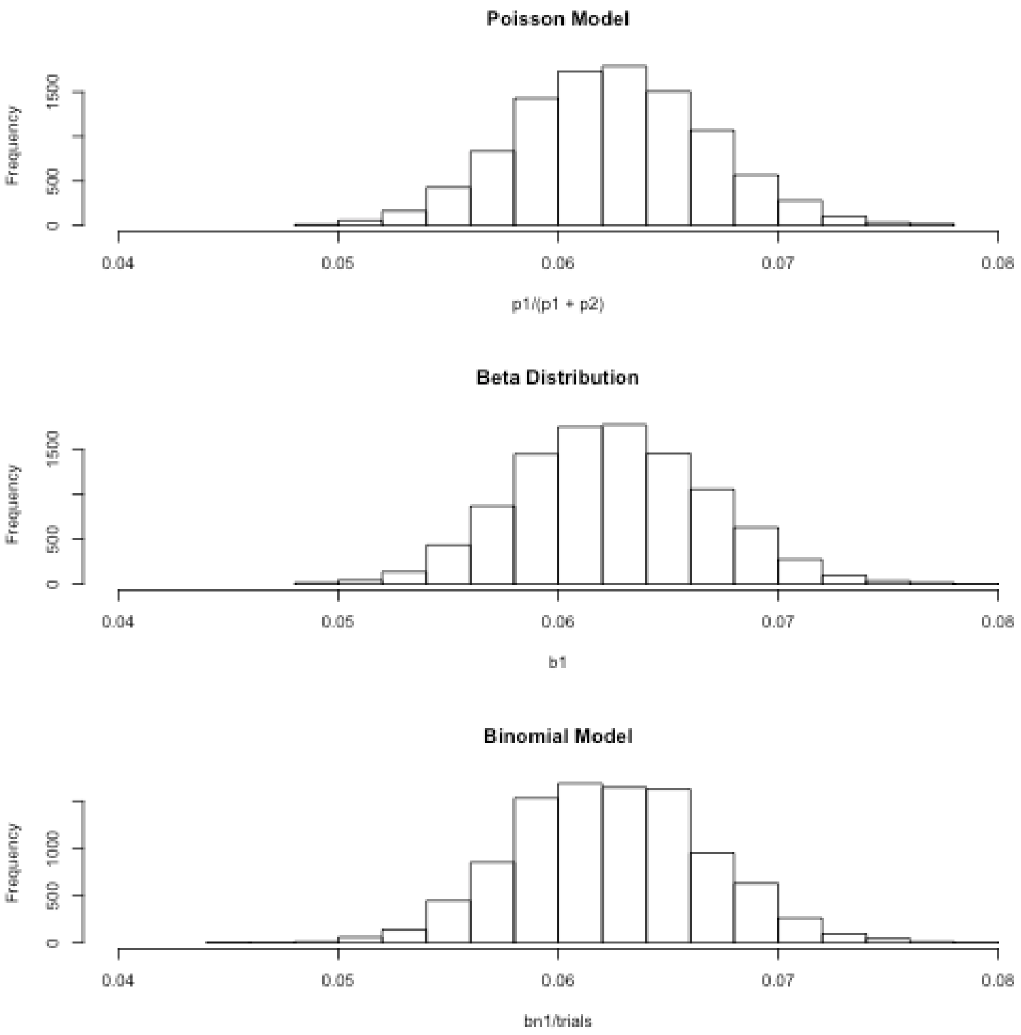

The histograms of these three processes show the good agreement of the distributions when X = 200 (meaning that the mean number of individuals of X is 200) and Y = 3000 (meaning that the mean number of other individuals in the community is 3000). These values of X and Y are likely small in comparison to real values from bacterial communities, and the agreement of the approximation improves with larger X and Y.

We repeat this simulation with a range of mean values for X to show how the CV changes when X comprises different proportions of the population. Again, we compare simulations from the ratio of Poisson variables and from a binomially-distributed variable.

```
#Create vectors to hold the proportion of X in the
commuinity, the CV from the Poisson model, and the CV from
the binomial model
seq.prop <- vector()
cv.loop.p <- vector()
cv.loop.b <- vector()

#Define a vector of mean abundances for X
start <- 10
end <- 1000
by <- 5

for(i in seq(start, end, by)){

num.samp <- 10000
X <- i
Y <- 3000
par(mfrow = c(3, 1))
p1 <- rpois(num.samp, X)
p2 <- rpois(num.samp, Y)

b1 <- rbeta(num.samp, X, Y)

#Compare the above distribution to data generated from
binomial distribution
trials <- X + Y
bn1 <- rbinom(num.samp, trials, X / (X + Y))

seq.prop[which(i == seq(start, end, by))] <- i / Y
cv.loop.p[which(i == seq(start, end, by))] <- cv(p1 /
(p1+p2))
cv.loop.b[which(i == seq(start, end, by))] <- cv(bn1 /
trials)
}

par(mfrow = c(2,1))
plot(seq.prop, cv.loop.p, xlab = "Proportion of community",
ylab = "CV", main = "Poisson Model")
plot(seq.prop, cv.loop.b, xlab = "Proportion of community",
ylab = "CV", main = "Binomial Model")
```

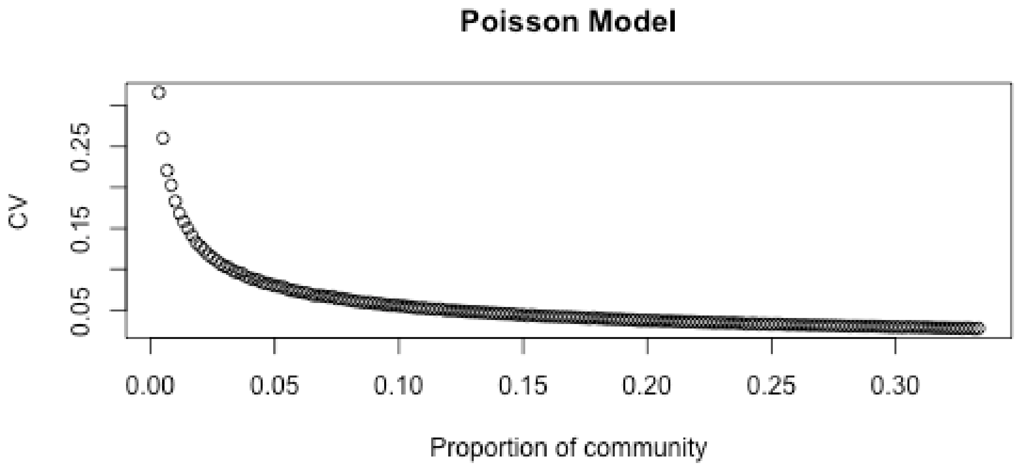

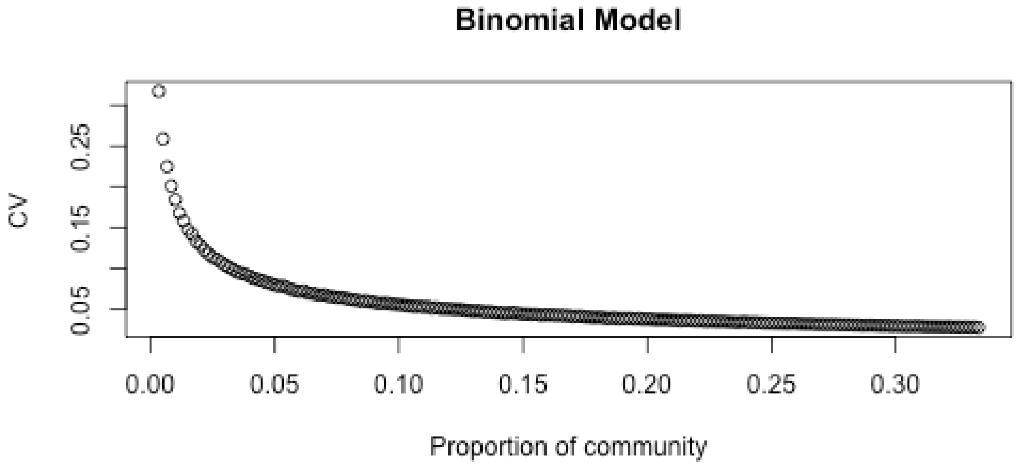

Thus, the distribution generated from taking the proportion of X in the overall community under the assumption of Poisson-distributed populations closely follows that of taking the proportion of binomially-distributed population in a community of fixed size. These two processes both generate the same patterns of changes in CV with varying mean relative abundance.
