# Supplementary material for: Environmental Disturbances Decrease the Variability of Microbial Populations within Periphyton
Source: mSystems. 2016 May 17;1(3):e00013-16. doi: 10.1128/mSystems.00013-16 (PMC5072133; doi:10.1128/mSystems.00013-16)
Supplement: Table S1 [file sys003162022st2.docx]

**Table S1:** Definitions of Community Properties And Their Relationships to Population Variability

|  | **Definition** | **Relation to Population Variability** |
| --- | --- | --- |
| **Coefficient of Variation (CV)** | The standard deviation of a population divided by the mean abundance of a population | Used as the metric of population variability in this study |
| **Predictability** | “the converse of uncertainty” (Colwell 1974); the degree to which a model can accurately forecast a desired parameter when relevant drivers are included in the model | Predictability of community composition increases when population variability decreases and when drivers of population variability are known. |
| **Stability** | A binary term (i.e. stable or unstable) that describes whether a community will return to its pre-disturbance composition after being changed as a result of disturbance (Pimm 1984) | In the absence of disturbance, stable communities should contain populations with lower spatial and temporal variability than populations in unstable communities. However, stable systems may exhibit a wide range of population variability, depending on the frequency and intensity of disturbance to the system. |
| **Resilience** | The rate at which a community returns to its pre-disturbance composition following perturbation (Pimm 1984) | There is no clear relation to population variability in the absence of disturbance, as resilience measures the rate of change in a community, rather than the magnitude of the deviation in community composition. However, if disturbance alters community properties, then highly resilient communities may only show the effects of disturbance briefly, due to their rapid rate of return to their prior state. |
| **Resistance** | The extent of compositional change in a community as a result of a disturbance (Pimm 1984) | Populations that are resistant to disturbance should show lower temporal variability, as their abundances are less affected by perturbation. However, there is no clear relationship between resistance and spatial variability when the disturbance regime is the same across space. |
