# Supplementary material for: Environmental Disturbances Decrease the Variability of Microbial Populations within Periphyton
Source: mSystems. 2016 May 17;1(3):e00013-16. doi: 10.1128/mSystems.00013-16 (PMC5072133; doi:10.1128/mSystems.00013-16)
Supplement: Table S2 [file sys003162022st3.docx]

**Table S2:** Comparison between theoretical and pseudo P values

| **Treatment** | AD | AS | DA | DD | DS | SA | SD | SS |
| --- | --- | --- | --- | --- | --- | --- | --- | --- |
| **Theoretical P-Value** | 0.0016  ** | 0.031  * | 0.033  * | 0.014  * | 0.0052  ** | 0.034  * | 0.0001  ** | 0.19 |
| **Pseudo**  **P-Value** | 0.008  ** | 0.037  * | 0.044  * | 0.023  * | 0.006  ** | 0.038  * | 0.000  ** | 0.158 |
